# Genomic characterization and immunotherapy for microsatellite instability-high in cholangiocarcinoma

**DOI:** 10.1186/s12916-024-03257-7

**Published:** 2024-01-29

**Authors:** Xu Yang, Baofeng Lian, Nan Zhang, Junyu Long, Yiran Li, Jingnan Xue, Xiangqi Chen, Yunchao Wang, Yanyu Wang, Ziyu Xun, Mingjian Piao, Chenpei Zhu, Shanshan Wang, Huishan Sun, Zhijian Song, Leilei Lu, Xiaowei Dong, Aodi Wang, Wenjin Liu, Jie Pan, Xiaorong Hou, Mei Guan, Li Huo, Jie Shi, Haohai Zhang, Jinxue Zhou, Zhenhui Lu, Yilei Mao, Xinting Sang, Liqun Wu, Xiaobo Yang, Kai Wang, Haitao Zhao

**Affiliations:** 1 Department of Liver Surgery, State Key Laboratory of Complex Severe and Rare Diseases, Peking Union Medical College Hospital, Chinese Academy of Medical Sciences and Peking Union Medical College, Beijing, China; 2 OrigiMed Co., Ltd, Shanghai, China; 3 Department of Breast Surgery, Peking, Union Medical College Hospital, Chinese Academy of Medical Sciences & Peking Union Medical College, Beijing, China; 4 Department of Radiology, Peking Union Medical College Hospital, Chinese Academy of Medical Sciences & Peking Union Medical College, Beijing, China; 5 Department of Radiotherapy, Peking Union Medical College Hospital, Chinese Academy of Medical Sciences & Peking Union Medical College, Beijing, China; 6 Department of Medical Oncology, Peking Union Medical College Hospital, Chinese Academy of Medical Sciences & Peking Union Medical College, Beijing, China; 7 Department of Nuclear Medicine, Peking Union Medical College Hospital, Chinese Academy of Medical Sciences & Peking Union Medical College, Beijing, China; 8 Department of Pathology, Peking Union Medical College Hospital, Chinese Academy of Medical Sciences & Peking Union Medical College, Beijing, China; 9 Center for Inflammation Research, Department of Anesthesia, Critical Care & Pain Medicine, Beth Israel Deaconess Medical Center, Harvard Medical School, Boston, MA USA; 10 Department of Hepatobiliary and Pancreatic Surgery, The Affiliated Cancer Hospital of Zhengzhou University & Henan Cancer Hospital, Zhengzhou, China; 11 Hepatobiliary and Pancreatic Surgery, Shenzhen Qianhai Shekou Free Trade Zone Hospital, Shenzhen, China; 12 Liver Disease Center, Affiliated Hospital of Qingdao University, Qingdao, China

**Keywords:** Microsatellite instability-high, Cholangiocarcinoma, PD-1 inhibitor, PD-L1 expression, Tumor mutation burden, Overall survival, Immune checkpoint inhibitor

## Abstract

**Background:**

Microsatellite instability-high (MSI-H) is a unique genomic status in many cancers. However, its role in the genomic features and immunotherapy in cholangiocarcinoma (CCA) is unclear. This study aimed to systematically investigate the genomic characterization and immunotherapy efficacy of MSI-H patients with CCA.

**Methods:**

We enrolled 887 patients with CCA in this study. Tumor samples were collected for next-generation sequencing. Differences in genomic alterations between the MSI-H and microsatellite stability (MSS) groups were analyzed. We also investigated the survival of PD-1 inhibitor-based immunotherapy between two groups of 139 patients with advanced CCA.

**Results:**

Differential genetic alterations between the MSI-H and MSS groups included mutations in *ARID1A*, *ACVR2A*, *TGFBR2*, *KMT2D*, *RNF43*, and *PBRM1* which were enriched in MSI-H groups. Patients with an MSI-H status have a significantly higher tumor mutation burden (TMB) (median 41.7 vs. 3.1 muts/Mb, *P* < 0.001) and more positive programmed death ligand 1 (PD-L1) expression (37.5% vs. 11.9%, *P* < 0.001) than those with an MSS status. Among patients receiving PD-1 inhibitor-based therapy, those with MSI-H had a longer median overall survival (OS, hazard ratio (HR) = 0.17, *P* = 0.001) and progression-free survival (PFS, HR = 0.14, *P* < 0.001) than patients with MSS. Integrating MSI-H and PD-L1 expression status (combined positive score ≥ 5) could distinguish the efficacy of immunotherapy.

**Conclusions:**

MSI-H status was associated with a higher TMB value and more positive PD-L1 expression in CCA tumors. Moreover, in patients with advanced CCA who received PD-1 inhibitor-based immunotherapy, MSI-H and positive PD-L1 expression were associated with improved both OS and PFS.

**Trial registration:**

This study was registered on ClinicalTrials.gov on 07/01/2017 (NCT03892577).

**Supplementary Information:**

The online version contains supplementary material available at 10.1186/s12916-024-03257-7.

## Background

The mismatch repair (MMR) pathway is essential for maintaining DNA replication fidelity, mutation avoidance, and genomic stability [1]. When the MMR pathway is not functioning correctly, cells are unable to correct errors during DNA replication, leading to an inconsistent number of microsatellite nucleotide repeats and instability in microsatellite regions [2]. This deficiency, known as deficient DNA mismatch repair (dMMR), is characterized by microsatellite instability-high (MSI-H) in tumors and has a unique genomic status and tumor microenvironments [3, 4]. Patients with MSI-H cancers, including gastrointestinal (GI) cancer, have experienced excellent effects from programmed cell death protein-1 (PD-1)-based immune checkpoint inhibitor (ICI) immunotherapy [5–7]. Based on this evidence, the Food and Drug Administration (FDA) approved programmed cell death protein-1 (PD-1) inhibitors monotherapy (and cytotoxic T lymphocyte-associated antigen 4 inhibitors) for patients with MSI-H/dMMR colorectal cancer and multiple other cancers, regardless of the location [3, 8–11].

Cholangiocarcinoma (CCA) is an aggressive and fatal cancer that originates in the bile duct epithelium [12, 13]. Unfortunately, the majority (> 65%) of patients with biliary tract cancer (BTC) have unresectable disease and patients with advanced CCA have a poor survival rate by classical systemic chemotherapy [12, 13]. However, recent advancements in next-generation sequencing (NGS) technologies have revealed genomic features that may help CCA patients receive precision medicine or immunotherapy [2, 12–16]. In particular, PD-1 inhibitor-based immunotherapy has shown encouraging efficacy in a subset of CCA. The Phase 3 TOPZA-1 and KEYNOTE-966 trials have demonstrated the survival benefits of adding PD-1/ligand 1 (PD-L1) inhibitors for patients with advanced CCA [17, 18]. The prevalence of MSI-H/dMMR in CCA is relatively low (1%-3%) [19–22], but these patients have shown promising efficacy for immunotherapy. Among advanced CCA patients with MSI-H/dMMR treated with the PD-1 inhibitor pembrolizumab in the Keynote-158 study could achieve a 40.9% objective response rate (ORR) [23], indicating that patients with MSI-H are highly sensitive to PD-1 inhibitors. Some case reports have also shown that MSI-H in patients with CCA predicts response to ICI therapy [24–26].

The impact of MSI-H status on the genomic profile and immunotherapy response in CCA is very important but has not been well characterized. In this study, we systematically analyzed the genomic characteristics and immunotherapy efficacy of MSI-H by comparing microsatellite stability (MSS) status in patients with CCA. Integrating histopathological features to identify potentially beneficiary patients for immunotherapy with a MSS status is also very important.

## Methods

### Patient characteristics

This study involved 887 patients who were diagnosed with histologically confirmed CCA and treated at the Peking Union Medical College Hospital (PUMCH) from 2017 to 2020 and The Affiliated Hospital of Qingdao University (QDUH) from 2017 to 2019. All MSI-H CCA patients were enriched by hand searches of medical records as much as possible to allow better comparability between the two groups. The study collected samples including tumor tissues and paired tumor-free tissues, and clinical data such as gender, age, tumor location, tumor size, tumor differentiation, lymph node metastasis, and tumor stage from electronic medical records. Before sample disposal, two independent pathologists reviewed all tumor tissues to confirm the pathological diagnoses. Only samples with an estimated tumor purity of > 20% on histopathological assessment were subjected to genomic profiling. The study obtained written informed consent from all participants and was approved by the Institutional Ethics Review Committee at PUMCH and QDUH (NCT03892577; RWSHBC). This study followed the guidelines of the Declaration of Helsinki and Good Clinical Practice.

### Genomic profiling

Genomic DNA was prepared from formalin-fixed, paraffin-embedded tumor samples and matched white blood cells (84.9%) or paracancerous/normal tissues (15.1%) using a DNA extraction kit (QIAamp DNA FFPE Tissue Kit), according to the manufacturer’s protocols. NGS-based cancer sequencing YuanSu (CSYS) panel (*n* = 824) or WES (*n* = 63) was performed as previously described in the OrigiMed [27–30]. Tumor samples were sequenced by CSYS panel with a mean coverage of 900 × and matched normal blood samples were sequenced to a mean unique coverage of 300 × . For WES, the mean coverage was 500 × for the tumor sample and 100 × for the matched normal blood samples on the Illumina NovaSeq6000 Platform (Illumina).

Genomic alterations, including single-nucleotide variations (SNVs), short and long insertions/deletions (indels), copy-number variations (CNVs), gene rearrangements, and gene fusions, were identified as follows: BWA (v0.6.2) was used for aligning raw reads to the human genome reference sequence (hg19); Picard (version 1.47) was used for removing PCR duplicates; MuTect (v1.7) was used for identifying SNVs; PINDEL (V0.2.5) was used for identifying indels polymorphisms; and SnpEff (v3.0) was used for annotating the functional impact of SNVs and indels. Control-FREEC (v9.7) was used to identify CNV regions. Gene fusions/rearrangements were detected using an in-house pipeline and assessed using Integrative Genomics Viewer (v2.4).

The study determined the functional significance of variants in genes by examining databases and published literature, including ClinVar, Catalogue of Somatic Mutations in Cancer, and PubMed. The study reported known or likely drivers and recurrent variants. Pathogenic mutations were defined as variants that clearly affect the function of a gene [30, 31]. Synonymous variants and variants with uncertain significance (VUS) (named VUS) were excluded because of lacking clinical significance [32]. Only pathogenic, likely pathogenic, and some meaningful mutations that are clear, potentially functionally, clinically significant, or functionally unknown were reported as clear somatic variants (named Not-VUS) in tumors.

The tumor mutation burden (TMB) was calculated by counting the total number of coding mutations per megabase as report [27, 33]. TMB values higher than 10 muts/Mb were considered TMB-high (TMB-H), and those less than 10 muts/Mb were considered TMB-low (TMB-L) [34]. The indel ratio is defined as the number of (frameshift) indel mutations per megabase in the target region [3, 35]. MSI status was determined based on the MANTIS score [36]. MSI-H is defined as more than 15% of selected microsatellite loci showing unstable in tumors compared to matched peripheral blood [29].

Fifteen cancer pathways (Additional File 1: Table S1), including phosphoinositide 3-kinase (PI3K), homologous recombination repair deficiency (HRD), wingless/integrated (WNT), fibroblast growth factor (FGF), cell cycle (CellCycle), switch/sucrose nonfermentable (SWI/SNF), base excision repair (BER), homologous recombination repair (HRR), MMR, nucleotide excision repair (NER), non-homologous end joining (NHEJ), Fanconi anemia (FA), checkpoint factor (CPF), translesion synthesis (TLS), and DNA damage response (DDR), were analyzed [26, 37].

### PD-1 inhibitor-based immunotherapy cohort of cholangiocarcinoma and efficacy evaluation

In patients who received PD-1 inhibitor-based therapy, follow-up was conducted to evaluate the efficacy and safety of the drugs until overall survival (OS) was determined. Further analysis was performed on patients who meet the following conditions: (1) patients should have an Eastern Cooperative Oncology Group (ECOG) performance status (PS) of 0–2 and normal baseline organ functions; (2) patients had at least one measurable lesion used to assess the therapeutic response according to the Response Evaluation Criteria in Solid Tumors (RECIST) version 1.1 [38]; (3) patients with radiologically or histologically confirmed advanced CCA who received PD-1 inhibitors based combination therapy with other agents of cancer treatment [39–41].

A total of 139 patients with advanced CCA who received PD-1 inhibitor-based combination therapy were enrolled in this study. PD-1 inhibitor-based therapy may have been heterogeneous for different PD-1 inhibitors (including pembrolizumab or nivolumab, toripalimab, camrelizumab, sintilimab, or tislelizumab every three weeks intravenously) and combination therapies in a real-world setting [39–41]. Patients were followed every six to eight weeks. The treatment effects were evaluated according to the RECIST version 1.1 guidelines [38] by professional radiologists at our center who were blinded to the therapeutic outcomes and clinicopathological features. Durable clinical benefit (DCB) was defined as complete response (CR), partial response (PR), or stable disease (SD) for ≥ 24 weeks; other was defined as no durable benefit (NDB) [42]. Progression-free survival (PFS) was defined as the time from initiating anti-PD-1 treatment to the first documented disease progression or death due to any causes, while OS was defined as the time between the start of anti-PD-1 treatment and death due to any causes.

### Identification and classification of potentially actionable alterations of cholangiocarcinoma

The OncoKB database was used to determine potentially actionable targets (PATs) of genetic alterations in druggable target [43]. All actionable alterations were classified as levels 1, 2, 3A/B, and 4 as follows. Level 1 means FDA-recognized biomarker predictive of response to an FDA-approved drug in this indication; while Level 2 means standard care biomarker recommended by the National Comprehensive Cancer Network (NCCN) or other professional guidelines predictive of response to an FDA-approved drug in this indication. Level 3A means compelling clinical evidence supports the biomarker as being predictive of response to a drug in this indication; while Level 3B means standard care or investigational biomarker predictive of response to an FDA-approved or investigational drug in another indication. For Level 4, this means there is compelling biological evidence to support that the biomarker can predict drug response [43]. Usually, the alterations of levels 1, 2, and 3A were deemed actionable targets [28, 30].

### Immunohistochemistry (IHC) analysis of PD-L1 and MMR expression

PD-L1 status was estimated by IHC staining of FFPE tissue sections using anti-PD-L1 antibodies clone 22C3 (DAKO, cat#m3666) or 28–8 (Abcam, Cat#ab205921) [29, 44]. The specimen was considered PD-L1 positive expression when the combined positive score (CPS) was either ≥ 1 [45–47]. IHC analysis was conducted to detect MMR-related proteins, including MLH1 (clone ES05, cat#IR079, DAKO), PMS2 (clone EP51, cat#IR087, DAKO), MSH2 (clone FE11, cat#IR08561-2, DAKO), and MSH6 (clone EP49, cat#IR086, DAKO). Tumors were classified as dMMR when the expression of at least one MMR protein was lost and as MMR-proficient when all four MMR proteins had positive nuclear staining in tumor cells [48].

### Statistical analysis

The study assessed normal distribution was assessed using the Kolmogorov–Smirnov test. Fisher’s exact test was utilized to compare categorical variables between multiple groups. For continuous variable comparisons between two groups, a two-tailed unpaired *t*-test was used when the data were normally distributed; otherwise, Wilcoxon’s test was used. The study identified variables associated with MSI-H using logistic regression analysis. For survival analysis, univariable and backward stepwise multivariable Cox proportional hazards regression models (if univariate *P* value of < 0.1) were used to calculate hazard ratios (HRs). Kaplan–Meier plots (log-rank tests) were used to describe the prognostic factors related to PFS and OS. The results are reported as HRs and 95% confidence intervals (CIs). The R package “survival” for survival analysis, “forestplot” for forest maps, and “ComplexHeatmap” for profiling heat maps were used. The function “somaticInteractions” from R package “maftools” was used to detect mutually exclusive or co-occurring mutations in MSI-H and MSS groups [49]. All reported *P* values were two-tailed, and *P* < 0.05 was considered statistically significant. All statistical analyses were performed using R version 4.1.1.

## Results

### Clinical characteristics of patients with CCA

A total of 887 patients with CCA were included in this study; 584 (65.8%) had intrahepatic cholangiocarcinoma (ICC) and 303 (34.2%) had extrahepatic cholangiocarcinoma (ECC). The median patient age was 60 years old (range: 19–89 years old). Among these patients, 60.2% were male, and 5.4% (48/887) were identified as MSI-H. The median TMB value was 3.7 (interquartile range [IQR]: 1.9–6.2) muts/Mb. The clinicopathological characteristics of the patients and individual details are shown in Additional File 2: Table S2 and Additional File 1: Table S3.

Compared with MSS patients, MSI-H patients had a significantly higher frequency of positive PD-L1 expression (CPS ≥ 1, 37.5% vs. 11.9%, *P* < 0.001) and frequency of TMB-H (100% vs. 8.7%, *P* < 0.001 by Fisher’s exact test) (Additional File 2: Table S2, Additional File 2: Figure S1). No statistically significant associations were observed between MSI status and clinical characteristics such as gender, age, tumor location, tumor size, tumor differentiation, and tumor stage (*P* > 0.05 by Fisher’s exact test).

### Genomic features of patients with CCA with different MSI statuses

In total, 7076 somatic SNVs and 2116 small indels, 272 fusions, and 1225 CNVs were identified. After removing VUS gene alternation, 4154 somatic SNVs, 1403 small indels, 169 fusions, and 993 CNV were retained for further analysis. As for 48 MSI-H tumors, 1176 somatic SNVs, 640 small indels, only 2 fusions, and 10 CNV were identified excluding VUS gene alternation (Additional File 1: Table S4).

**Fig. 1:**
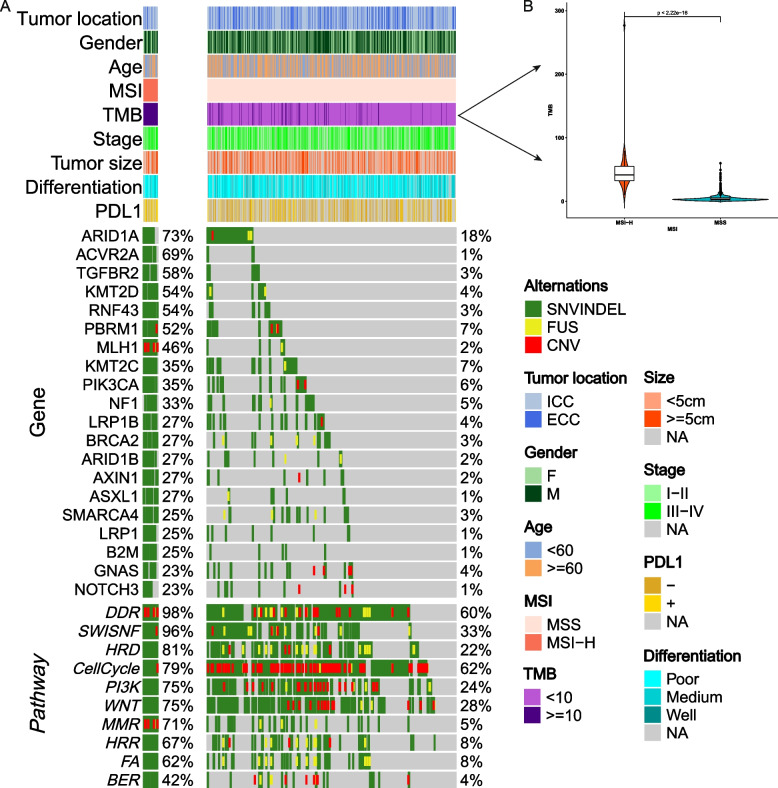
Genomic mutation landscape in cholangiocarcinoma distinguished by MSI status. **A** Genetic profile of significantly altered genes and associated clinicopathological characteristics of all 887 cholangiocarcinoma patients. Only the top 20 altered genes and top 10 pathways of the MSI-H group are shown. **B** The TMB value in the MSI-H group was significantly higher than that in the MSS group. MSI-H, microsatellite instability-high; MSS, microsatellite stability; TMB, tumor mutation burden

The differential gene alterations significant between the MSI-H and MSS groups were *ARID1A* (73% vs. 18%), *ACVR2A* (69% vs. 1%), *TGFBR2* (58% vs. 3%), *KMT2D* (54% vs. 4%), *RNF43* (54% vs. 3%), *PBRM1* (52% vs. 7%), *MLH1* (46% vs. 2%), *KMT2C* (35% vs. 7%), *PIK3CA* (35% vs. 6%), and *NF1* (33% vs. 5%) (Fig. 1A, Additional File 1: Table S5). We also found that the mutated genes *ARID1A*, *ACVR2A*, *TGFBR2*, *RNF43*, *KMT2D*, and *PBRM1* were enriched with the MSI-H status of patients (Additional File 2: Figure S2). Among 15 pathways in cancer, the differential mutation pathways between the MSI-H and MSS groups included the DDR (98% vs. 60%), SWI/SNF (96% vs. 33%), HRD (81% vs. 22%), CellCycle (79% vs. 62%), and PI3K (75% vs. 24%) (Additional File 1: Table S6, Fig. 1A). In addition, tumors with an MSI-H status had a higher TMB (median 41.7 vs. 3.1 Mut/Mb, *P* < 0.001; Fig. 1B) and a higher insertion indel ratio (median 0.1698 vs. 0.0588, *P* < 0.001 by Wilcoxon’s test) compared with those with MSS (Additional File 2: Figure S3).

### Association between the efficiency of PD-1 inhibitor-based treatment and MSI status

In this cohort, 139 CCA patients underwent PD-1 inhibitor-based immunotherapy (NCT03892577). Among them, 16 (11.5%) had tumors with MSI-H (*N* = 12) or dMMR (*N* = 4). Three of the four dMMR patients had MLH1 and PMS2 loss, and the one had MSH2 and MSH6 loss. The median age of the 139 patients was 61 years (range, 30–84 years), and 58.3% were male. 107 (77.0%) were ICC and 32 (23.0%) were ECCs. The maximum tumor diameter ≥ 5 cm was 55 (39.6%) patients. The ECOG PS scores of 0, 1, and 2 were 31.7%, 51.8%, and 16.5%, respectively. The proportions of TNM stage I, II, III, and IV were 2.9%, 11.5%, 32.4%, and 53.2%, respectively. The percentages of patients with PD-L1 CPS ≥ 1 and PD-L1 CPS ≥ 5 were 44.6% and 29.5%, respectively (Additional File 2: Table S7). Chemotherapy (*N* = 24, 17.3%), lenvatinib (*N* = 82, 59.0%), apatinib (*N* = 9, 6.5%), and other drugs (*N* = 24, 17.3%) were used in combination with PD-1 inhibitors. The proportions of patients treated with pembrolizumab, nivolumab, toripalimab, camrelizumab, and other PD-1 inhibitors were 31.7%, 13.7%, 28.1%, 15.1%, and 11.4%, respectively. Compared with the MSS group in the immunotherapy cohort, the MSI-H group had better ECOG PS scores, more PD-L1 CPS ≥ 5, more combinations of PD-1 inhibitors combined with chemotherapy, more stage IV, and more TMB-H (*P* < 0.05 by Fisher’s exact test).

**Fig. 2:**
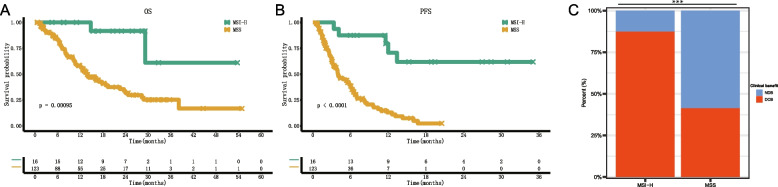
Kaplan‒Meier estimates of overall survival (**A**), progression-free survival (**B**), clinical benefit histogram (**C**) of 139 advanced cholangiocarcinoma patients receiving PD-1 inhibitor-based therapy stratified by MSI status. MSI-H, microsatellite instability-high; MSS, microsatellite stability

The median follow-up of the 139 patients was 14.1 (IQR: 7.7–27.4, range 1.07–70.3) months. As of the last follow-up, 100 (71.9%) patients had died, 39 (28.1%) patients were censored, 37 of them were still alive, and two patients were lost to follow-up. The median OS was 14.8 (95% CI: 12.6–16.9) months. We observed longer median OS (not evaluable [NE] vs. 13.5 m, HR = 0.17 (95% CI: 0.06–0.46, *P* = 0.001) and longer median PFS (NE vs. 4.0 m, HR = 0.14 (95% CI: 0.05–0.34, *P* < 0.001) in MSI-H patients compared with MSS patients by log-rank tests (Fig. 2A and B). There were 65 (46.8%) patients who reached DCB after PD-1 inhibitor combination therapy, including 87.5% MSI-H patients and 41.5% MSS patients (OR = 2.11, *P* < 0.001 by Fisher’s exact test; Fig. 2C). The ORR (68.8% vs. 17.9%, OR = 3.84, *p* < 0.001) and disease control rate (DCR) (100.0% vs. 65.9%, OR = 1.52, *P* = 0.003 by Fisher’s exact test) were also higher in the MSI-H group than the MSS group (Additional File 2: Figure S4).

### Univariate and multivariate survival analyses of the CCA cohort receiving PD-1 inhibitor-based therapy

Due to the small number of MSI-H patients, potential benefits from histopathological and clinical factors should be identified to confirm the efficacy of immunotherapy for patients with advanced CCA.

Eleven potential prognostic variables for OS were selected based on univariate Cox analysis, including age, largest tumor size, ECOG PS, treatment line, combination agents, PD-L1 (CPS ≥ 1), PD-L1 (CPS ≥ 5), MSI status, TMB (≥ 10 muts/Mb), SWI/SNF pathway, and MMR pathway (Table 1). In the multivariate analysis by backward stepwise multivariable Cox proportional hazards regression models, ECOG PS (1–2 vs. 0: HR, 3.096; 95% CI, 1.832–5.263, *P* < 0.001) and treatment line (≥ 2 vs. 0–1: HR,2.247; 95% CI, 1.289–3.922, *P* = 0.004), largest tumor size (≥ 5 cm vs. < 5 cm: HR,1.635; 95% CI, 1.048–2.552, *P* = 0.030) were independently associated with a significantly shorter OS. Conversely, PD-L1 expression (CPS ≥ 5 vs. < 5: HR, 0.424; 95% CI, 0.259–0.694, *P* = 0.012), combination therapy (chemotherapy vs. target therapy: HR,0.403; 95% CI, 0.191–0.850, *P* = 0.017) and MSI status (MSI-H vs. MSS: HR, 0.265; 95% CI, 0.093–0.754, *P* = 0.013) were associated with a significantly longer OS (Table 1).

**Table 1:** Univariate and multivariate analyses of prognostic factors for progression-free survival (PFS) and overall survival (OS) in patients with advanced cholangiocarcinoma

Variates	Univariate analysis for PFS	Multivariate analysis		Univariate analysis for OS	Multivariate analysis	
	***P* value**	***P* value**	**HR (95% CI)**	***P* value**	***P* value**	**HR (95% CI)**
Age (< 60 vs. ≥ 60)	0.554			**0.026**		
Gender (female vs. male)	0.740			0.739		
Tumor location (ICC vs. ECC)	0.139			0.462		
Largest tumor size (≥ 5 cm vs. < 5 cm)	0.157			**0.009**	**0.030**	1.635 (1.048–2.552)
ECOG-PS (1–2 vs. 0)	** < 0.001**	0.063	1.618 (0.974–2.688)	** < 0.001**	** < 0.001**	3.096 (1.832–5.263)
Treatment line (≥ 2 vs. 0–1)	0.344			**0.007**	**0.004**	2.247 (1.289–3.922)]
Combination agents (Chemotherapy vs. target therapy)	**0.026**			**0.006**	**0.017**	0.403 (0.191–0.850)
PD-L1 (CPS ≥ 1 vs. < 1)	**0.002**			**0.031**		
PD-L1 (CPS ≥ 5 vs. < 5)	** < 0.001**	**0.004**	0.467 (0.278–0.784)	**0.001**	**0.001**	0.424 (0.259–0.694)
MSI (MSI-H vs. MSS)	** < 0.001**	**0.002**	0.198 (0.069–0.563)	**0.001**	**0.013**	0.265 (0.093–0.754)
TNM stage (IV vs. I–III)	**0.048**			0.103		
T stage (T3–T4 vs. T1–T2)	0.946			0.815		
N stage (N1–N2 vs. N0)	0.435			0.199		
M stage (M1 vs. M0)	**0.048**			0.103		
TMB (≥ 10 vs. < 10 muts/Mb)	**0.002**			**0.012**		
SWI/SNF pathway (Mut vs. WT)	**0.004**			**0.026**		
MMR pathway (Mut vs. WT)	**0.008**			**0.016**		
WNT pathway (Mut vs. WT)	**0.012**			0.228		

Abbreviations: *CI* Confidence interval, *CPS* Combined positive score, *ECC* Extrahepatic cholangiocarcinoma, *ECOG-PS* Eastern Cooperative Oncology Group-performance status, *HR* Hazard ratio, *ICC* Intrahepatic cholangiocarcinoma, *MMR* Mismatch Repair, *MSI-H* Microsatellite instability-high, *MSS* Microsatellite stability, *Mut* Mutation, *OS* Overall survival, *PFS* Progression-free survival, *PD-L1* Programmed death ligand 1, *SWI/SNF* Switch/sucrose nonfermentable, *TMB* Tumor Mutation Burden, *WNT* Wingless/Integrated, *WT* Wild type

The median PFS was 5.3 (95% CI, 3.7–6.9) months. Multivariate Cox analysis showed that only PD-L1 expression (CPS ≥ 5 vs. < 5: HR,0.467; 95% CI, 0.278–0.784, *P* = 0.004) and MSI status (MSI-H vs. MSS: HR, 0.198; 95% CI, 0.069–0.563, *P* = 0.002) were independent predictors of longer PFS (Table 1).

### Guidance of MSI-H combined with PD-L1 expression in the immunotherapy of CCA patients

**Fig. 3:**
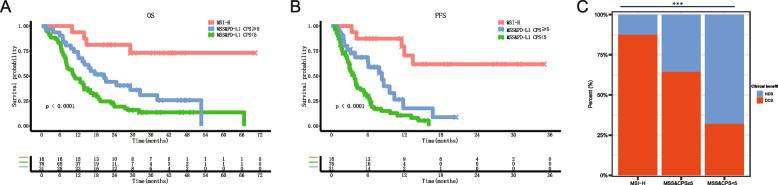
Kaplan–Meier curves for overall survival, progression-free survival and clinical benefit histogram of three classifications stratified according to MSI-H combined with PD-L1 CPS ≥ 5 (**A**–**C**). MSI-H, microsatellite instability-high; MSS, microsatellite stability; Mut, mutation; NDB, no durable benefit; OS, overall survival; PFS, progression-free survival; PD-L1, programmed death ligand 1; TMB, tumor mutation burden; WT, wild type

Among the 139 CCA patients receiving PD-1 inhibitor-based immunotherapy, 29.5% (41/139) harbored high PD-L1 expression (CPS ≥ 5). Patients in the PD-L1 CPS ≥ 5 expression group had longer OS, PFS, and higher DCB rates than those in the PD-L1 CPS < 5 expression group (Additional File 2: Figure S5, ABC). Integrating PD-L1 expression (CPS ≥ 5) and MSI status, we found that the OS of the MSI-H, MSS-PD-L1CPS ≥ 5, and MSS-PD-L1 CPS < 5 groups were NE, 20.6 months, and 10.6 months (*P* < 0.001 by log-rank tests, Fig. 3A), respectively; the PFS of these groups were NE, 8.3 months, and 3.8 months (*P* < 0.001 by log-rank tests, Fig. 3B), respectively. Their DCB rates were 87.5%, 64.5%, and 32.1% (*P* < 0.001 by Fisher’s exact test, Fig. 3C), respectively. It showed the MSI-H status combined with PD-L1 expression could well distinguish the efficacy of immunotherapy.

### Factors contributing to MSI-H status

**Fig. 4:**
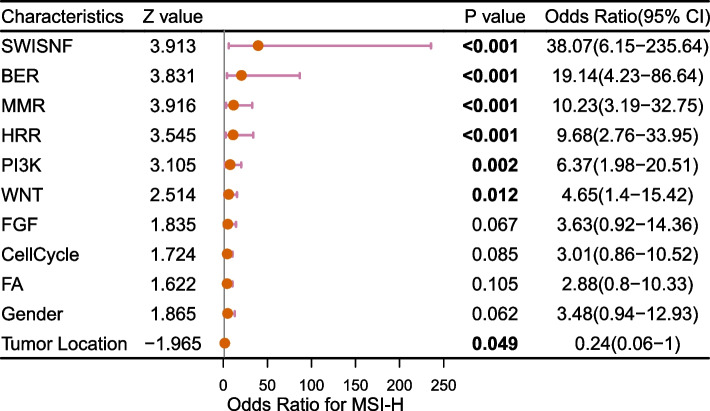
Factors contributing to MSI-H status. Using multivariate logistic regression, the association between pathways and clinical factors, and MSI-H status were analyzed. MSI-H: microsatellite instability-high

We analyzed the association between mutations of 15 pathways and important clinicopathological parameters (like age, gender, tumor location), and MSI-H status, to analyze what factors contribute to MSI-H status. Results showed that the SWI/SNF (OR = 38.07), BER (OR = 19.14), MMR (OR = 10.23), HRR (OR = 9.68), PI3K (OR = 6.37), and WNT (OR = 4.65) pathways have contributed to MSI-H status by multivariate logistic regression (*P* < 0.05). Meanwhile, tumor location of ICC (OR = 0.24, *P* = 0.049) had borderline contributed to MSI-H status (Fig. 4). When seeing the SWI/SNF, BER, MMR, and HRR pathway mutation, we may reflect a positive correlation with MSI-H status.

### Potentially actionable targets (PATs) and co-altered mutations in CCA with different MSI statuses

**Fig. 5:**
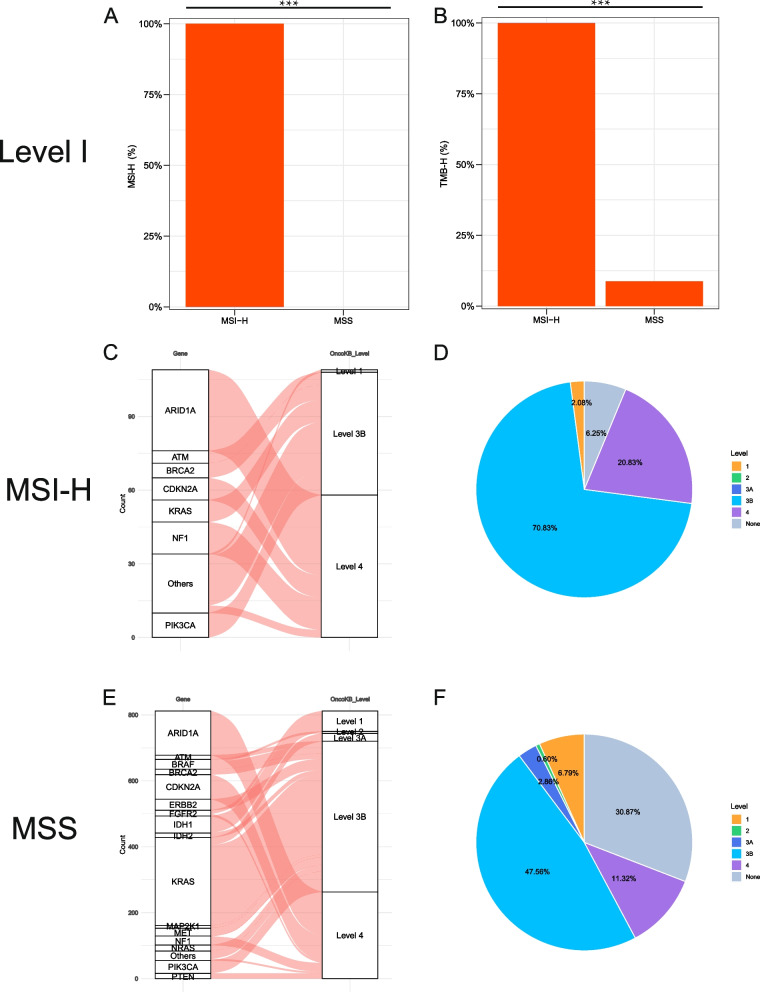
Potentially actionable alterations in MSI-H and MSS cancer based on the OncoKB database. **A**, **B** Level I of the proportions of MSI-H (100% vs. 0%) and TMB-H (100% vs. 8.7%) in MSI-H and MSS cancer. **C**, **E** Flow diagram in the upper part shows the list of translational targets for each OncoKB recommendation level in MSI-H, and the lower part presents the MSS cholangiocarcinoma. The widths of the belts indicate different frequencies for each target at every level. **D**, **F** The upper and lower pie plot shows the distribution of OncoKB levels for translational targets in patients with MSI-H or MSS cholangiocarcinoma. MSI-H, microsatellite instability-high; MSS, microsatellite stability; TMB-H, tumor mutation burden-high

We used the OncoKB database to annotate the PATs and found that all the MSI-H groups had level I evidence of pembrolizumab use; TMB-H was also considered to have level I evidence of pembrolizumab use. So, only 8.7% of patients in the MSS group (with TMB-H) fulfilled level I evidence for the use of pembrolizumab (Fig. 5A and B). Except for the targets of MSI-H and TMB-H, only 2.08% of patients had a PAT gene mutation (≤ level 3A) in the MSI-H group, compared with 10.25% in the MSS group (Fig. 5C and D). In the MSS group, *IDH1*, *KRAS*, *BRAF* mutation, and *FGFR2* fusion were the main target genes (Fig. 5E and F).

We further explored the underlying differences of co-altered mutations in MSI-H and MSS groups. Co-occurrence mutation statuses in the MSI-H group were observed between *ARID1A:NOTCH3*,* ARID1A:KRAS*,* KMT2D:LRP1B*,* MSH3:OBSCN*, and *ATM:POLE*. However, *NF1:LRP1B* was identified exclusively. On the other hand, in the MSS group, *TP53:CDKN2A*,* TP53:TERT*,* TP53:FBXW7*,* KRAS:SF3B1*, and *KRAS:CDKN2A* were identified co-occurrence; however, *TP53* mutants with *STK11*, *ATM*, *IDH1*, or *BAP1* mutations; and *KRAS* mutation with *ERBB2*,* BRAF*,* NF1*,* IDH1*,* BAP1*, or *TERT* mutations were identified exclusively (Additional File 2: Figure S6).

## Discussion

Our study comprehensively investigated the genomic alterations and PATs for precision medicine according to MSI status in 887 patients with CCA. Tumors with MSI-H status were associated with a higher TMB value and more positive PD-L1 expression but fewer PATs. Furthermore, by investigating immunotherapy in 139 advanced CCA patients, we found that MSI-H patients had longer PFS and OS than MSS patients receiving PD-1 inhibitor-based therapy. By integrating MSI-H with positive PD-L1 expression, we identified certain patients with advanced CCA who may benefit from PD-1 inhibitor-based immunotherapy.

MSI-H cancers have distinct genomic features, and this study chose non-VUS gene alterations for further analysis to emphasize clinical translational precision medicine. The mutation frequencies of *ARID1A*, *ACVR2A*, *TGFBR2*, *KMT2D*, and *RNF43* were significantly higher in the MSI-H group of our cohort. We found that the TMB value of the MSI-H group was higher than those of the MSS group. Abnormal pathways, including the DDR, SWI/SNF, and HRD, were highly frequent (> 80%) in the MSI-H group. Similarly, Eluri et al. reported that the frequency of MSI-H in 7565 ICC cases was 1.8%, and the median TMB of MSI-H patients was 21.7 muts/Mb. Meanwhile, the study also showed that genomic alterations in *TP53* (59.9%), *PRBM1* (37.2%), *ARID1A* (13.9%), and *APC* (13.9%) were enriched in patients with MSI-H [50]. In another study, *RNF43* and *KMT2D* mutations frequently occurred in CCA patients with MSI-H and TMB-H status [51]. Similarly, an analysis of the genomic landscape of MSI-H in 11,395 tumors across 30 cancer types showed that mutations in *ACVR2A* (73%), *KMT2D* (68%), *KMT2B* (66%), and MMR-related genes were enriched in the MSI-H group [52].

Tumors with MSI-H have dysfunctions that correct errors introduced in microsatellites, which could lead to more frameshift mutations, higher TMB value, and increased neoantigens formation and tumor-specific antigens which are thought to be unknown and novel to the individual immune system [53]. Then, tumors with MSI-H are highly infiltrated with cytotoxic T-cell lymphocytes like CD8 + T-cells with a Tc1 phenotype and CD4 + T-cells with a Th1 phenotype, to raise tumor-specific immune responses, which leads to sensitivity to ICI of MSI-H tumors [54]. Some studies have found that MSI-H in patients with CCA has promising efficacy in immunotherapy, however, the patients’ numbers were small [21, 23–26, 50, 55, 56]. In our cohort with 139 patients, we found that CCA patients with MSI-H had longer median OS (HR = 0.17, *P* = 0.001) and PFS (HR = 0.14, *P* < 0.001) than CCA patients with MSS, indicating that patients with MSI-H may have more opportunities to benefit from immunotherapy. In the KEYNOTE-158 study, 22 advanced CCA patients with MSI-H/dMMR received pembrolizumab treatment and ORR could reach up to 40.9%, with median PFS and median OS of 4.2 (2.1–24.9) and 19.4 (6.5-NR), respectively; ≥ 3 years OS rate exceeded 30% [23]. However, for the 104 advanced BTC patients with MSS status that received pembrolizumab treatment, the ORR was only 5.8%, with median PFS and median OS of only 2.0 (1.9–2.1) and 7.4 (5.5–9.6) months, respectively; the survival rate of ≥ 3 years was only about 10% [57]. These results suggested that patients with MSI-H have more opportunities to benefit from immunotherapy. In our study, MSI-H status was a protective factor for both OS and PFS of immunotherapy in multivariate analysis. However, another study found that MSI-H (*N* = 15) was not an independent protective factor (*P* = 0.162) in refractory CCA with PD-L1 positivity (CPS≥ 1 or tumor proportion score (TPS) ≥ 1%) and treated with pembrolizumab, indicating that PD-L1 expression is also another important confounding factor [58].

Our study found that positive PD-L1 expression was relatively high in patients with MSI-H status. In many types of GI cancers, including BTC, colorectal cancer, and gastric cancer, PD-L1 expression was significantly associated with MSI-H status (*P* < 0.001) [22, 59, 60]. In our study, we observed that the combination of MSI status and PD-L1 expression could distinguish the prognosis of immunotherapy for both OS and PFS. We found patients with PD-L1 CPS ≥ 5 had a better both OS and PFS than patients with CPS < 5 in multivariate analysis, which is consistent with our previous study [61–64]. However, the significance of PD-L1 expression as a biomarker for BTC immunotherapy remains controversial. Yoon et al. reported that a PD-L1 CPS ≥ 1 does not distinguish well from the immunotherapy response in BTC [45]. Another study showed that the median PFS and OS of BTC patients did not differ according to the CPS cutoff values (1, 5, and 10) (*P* > 0.05 for all) [47]. Zhou et al. found a correlation between PD-L1 expression in patients with CPS ≥ 5 and CPS < 5 and the efficacy of anlotinib along with the PD-1 inhibitor; but they observed a similar median PFS (6.80 vs. 6.24 months, *P* > 0.05) [62]. In another prospective trial, the high PD-L1 expression (CPS ≥ 5) group did not show any differences in median PFS compared with the low PD-L1 expression group (high vs. low: 5.0 vs. 6.4 months, *p* = 0.81) [65]. Therefore, although patients with PD-L1 CPS ≥ 5 may benefit from PD-1 inhibitor immunotherapy for both OS and PFS in our cohort, the significance of PD-L1 expression and cut-off values still need to be confirmed in prospective and large retrospective cohorts.

We found that the TMB value of the MSI-H group was significantly higher than that of the MSS group, which is a consensus and consistent with the results of other studies involving many cancers [66]. In addition, we also found more indel alterations in the MSI-H CCA group, similar to those observed in colorectal cancer [67]. Meanwhile, our results also showed that mutated pathways, such as SWI/SNF, BER, MMR, and HRR, mostly contributed to the MSI-H status. Many studies have found a high frequency of MSI-H mutations in the SWI/SNF pathway, including intestinal and pancreatic cancers [68–70]. Wang et al. defined co-mutations (combinations of HRR-BER and HRR-MMR) in the DDR pathway and found that co-mutated DDR was significantly associated with MSI-H and TMB-H status [37]. Mutations in the MMR pathway surely contribute to the MSI-H status [53]. Similarly, Zhou et al. found that the percentage of MSI-H patients in the HRR-mut group was higher than that in the HRR-WT group in patients with colon cancer [71]. However, in the multivariate Cox analysis of immunotherapy efficacy in our cohort, the prognostic values of TMB, SWI/SNF pathway, or MMR pathway mutations were not significant (*P* > 0.05).

The percentage of altered genes that can be used as therapeutic targets (level ≤ 3A) according to the OncoKB database in the MSI-H group is low, while the percentage of altered genes in MSS group for potential therapeutic targets could be up to 10%, mainly including *IDH1* (ivosidenib), *KRAS* (adagrasib), *FGFR2* fusion (infigratinib), and *BRAF* mutation (dabrafenib plus trametinib). It showed the spectrum of precision medicine is different between the two groups. Nearly 8.7% of the patients belonged to the TMB-H group in the MSS group, while all (100%) of the MSI-H patients belonged to the TMB-H group, which can also be recommended to use the PD-1 inhibitor pembrolizumab (level I). Similarly, Eluri et al. also observed that MSI-H cases had lower frequencies of previously identified, actionable ICC drivers, such as *FGFR2* fusions (0% vs. 9%, *p* = NS), *IDH1* (3.7% vs. 14.5%, *p* < 0.0001), and *IDH2* (0% vs. 4.1%, *p* = 0.007) [50]. Different gene alterations and co-altered patterns may partly explain the low prevalence of PAT for other precision medicines except for ICI in the MSI-H CCA group [51]. MSI-H patients with CCA seem only amenable to ICI-based therapy.

Our study provided a large cohort and a great resource to comprehensively investigate genomic alterations and immunotherapy in patients with CCA. However, this study has some limitations. First, from a retrospective perspective, the “enrichment” process of patients with MSI-H tumors may have overestimated the proportion of the natural prevalence of MSI-H. However, this study focused on assessing genomic features and comparing immunotherapy efficacy between the two groups, so we wanted to enroll as many MSI-H patients as possible. Second, we just compared DNA-level characteristics according to MSI status, we hope to analyze the multi-omics feature (including RNA sequencing and immune marker by IHC) between MSI-H and MSS groups further. Third, in our immunotherapy cohort, the heterogeneity of PD-1 inhibitor-based therapy may stem from variations across different PD-1 inhibitors and combination therapies. But we found MSI status and PD-L1 CPS ≥ 5 could stratify the cohort prognosis in log-rank tests and multivariable Cox analysis. We encourage validating our findings in other large cohorts.

## Conclusions

The proportion of MSI-H in CCA was low. The mutation spectrum was different between MSI-H and MSS status. The MSI-H status of patients with CCA was associated with TMB-H and positive PD-L1 expression. Moreover, MSI-H and positive PD-L1 expression were associated with improved both OS and PFS in patients with advanced CCA who received PD-1 inhibitor-based immunotherapy.

### Supplementary Information

**Additional file 1: Table S1.** Fifteen pathways in cancer. **Table S3.** Individual Clinicopathological characteristics of 887 patients with cholangiocarcinoma. **Table S4.** Genomic alterations in 48 patients with MSI-H cholangiocarcinoma. Table S5. Different gene alterations in MSI-H and MSS cancer. **Table S6.** Different pathway alterations in MSI-H and MSS cancer. 

**Additional file 2: Table S2.** Clinicopathological characteristics of 887 patients with cholangiocarcinoma. **Figure S1.** Representative cases of hematoxylin-eosin staining and high PD-L1 expression of MSI-H patients with cholangiocarcinoma. (ABEF) Two ICC cases, (CDGH) 2 ECC cases. ECC: extrahepatic cholangiocarcinoma; ICC: intrahepatic cholangiocarcinoma. **Figure S2.** Proportion of mutated cholangiocarcinoma samples by MSI status: (y-axis) proportion in MSI-H-specific gene; (x-axis) proportion in MSS-specific gene. MSI-H: microsatellite instability-high, MSS: microsatellite stability. **Figure S3.** The indel ratio of cholangiocarcinoma in MSI-H and MSS cancer. MSI-H: microsatellite instability-high, MSS: microsatellite stability. **Figure S4.** ORR (A) and DCR (B) of 139 advanced cholangiocarcinoma patients receiving PD-1 inhibitor-based therapy stratified by MSI status. DCR: disease control rate, MSI: microsatellite instability, ORR: Objective Response Rate. **Figure S5.** Kaplan‒Meier estimates of overall survival and progression-free survival and clinical benefit histogram of patients with advanced cholangiocarcinoma receiving PD-1 inhibitor-based therapy stratified by PD-L1 CPS ≥5 (ABC). PD-L1: programmed death ligand 1; MSI-H: microsatellite instability-high; MSS: microsatellite stability; Mut: mutation; NDB: no durable benefit; OS, overall survival; PFS, progression-free survival; PD-L1: programmed death ligand 1; TMB: tumor mutation burden. **Figure S6.** The spectrum of top 30 co-occurring or exclusively occurring mutations for MSI-H (A) and MSS (B) cholangiocarcinoma. MSI-H: microsatellite instability-high, MSS: microsatellite stability. **Table S7.** Baseline clinicopathological characteristics of 139 patients with advanced cholangiocarcinoma who received PD-1 inhibitor-based therapy.

## Data Availability

All data supporting the findings of this study are available in this article and its online supplementary material files. Further inquiries can be directed to the corresponding author.

## Abbreviations

BER: Base excision repair
BTC: Biliary tract cancer
CCA: Cholangiocarcinoma
CellCycle: Cell cycle
CI: Confidence interval
CNV: Copy-number variation
CPF: Checkpoint factor
CPS: Combined positive score
CR: Complete response
CSYS: Cancer sequencing YuanSu
DCB: Durable clinical benefit
DCR: Disease control rate
DDR: DNA damage response
dMMR: Deficient DNA mismatch repair
ECC: Extrahepatic cholangiocarcinoma
ECOG-PS: Eastern Cooperative Oncology Group-Performance Status
FA: Fanconi anemia
FDA: Food and Drug Administration
FGF: Fibroblast growth factor
GI: Gastrointestinal
HR: Hazard ratio
HRD: Homologous recombination repair deficiency
HRR: Homologous recombination repair
ICC: Intrahepatic cholangiocarcinoma
ICI: Immune checkpoint inhibitor
Indels: Insertions/deletions
IQR: Interquartile range
MMR: Mismatch repair
MSI-H: Microsatellite instability-high
MSS: Microsatellite stability
Mut: Mutation
NA: Not available
NCCN: National Comprehensive Cancer Network
NDB: No durable benefit
NE: Not evaluable
NER: Nucleotide excision repair
NGS: Next-generation sequencing
NHEJ: Non-homologous end joining
NSCLC: Non-small cell lung cancer
OR: Odd ratio
ORR: Objective response rate
OS: Overall survival
PAT: Potentially actionable target
PD: Progressive disease
PD-1: Programmed death 1
PD-L1: Programmed death-ligand 1
PFS: Progression-free survival
PI3K: Phosphoinositide 3-kinase
PR: Partial response
PUMCH: Peking Union Medical College Hospital
QDUH: Qingdao University
RECIST: Response Evaluation Criteria in Solid Tumors
SD: Stable disease
SNV: Single-nucleotide variation
SWI/SNF: Switch/sucrose nonfermentable
TLS: Translesion synthesis
TMB: Tumor mutation burden
TNM: Tumor node metastasis
TPS: Tumor proportion score
UK: Unknow
VUS: Variants with uncertain significance
WNT: Wingless/integrated
WT: Wild type
