# Supplementary material for: Genomic characterization and immunotherapy for microsatellite instability-high in cholangiocarcinoma
Source: BMC Med. 2024 Jan 29;22:42. doi: 10.1186/s12916-024-03257-7 (PMC10823746; doi:10.1186/s12916-024-03257-7)
Supplement: Supplementary file 2 — Additional file 2: Table S2. Clinicopathological characteristics of 887 patients with cholangiocarcinoma. Figure S1. Representative cases of hematoxylin-eosin staining and high PD-L1 expression of MSI-H patients with cholangiocarcinoma. (ABEF) Two ICC cases, (CDGH) 2 ECC cases. ECC: extrahepatic cholangiocarcinoma; ICC: intrahepatic cholangiocarcinoma. Figure S2. Proportion of mutated cholangiocarcinoma samples by MSI status: (y-axis) proportion in MSI-H-specific gene; (x-axis) proportion in MSS-specific gene. MSI-H: microsatellite instability-high, MSS: microsatellite stability. Figure S3. The indel ratio of cholangiocarcinoma in MSI-H and MSS cancer. MSI-H: microsatellite instability-high, MSS: microsatellite stability. Figure S4. ORR (A) and DCR (B) of 139 advanced cholangiocarcinoma patients receiving PD-1 inhibitor-based therapy stratified by MSI status. DCR: disease control rate, MSI: microsatellite instability, ORR: Objective Response Rate. Figure S5. Kaplan‒Meier estimates of overall survival and progression-free survival and clinical benefit histogram of patients with advanced cholangiocarcinoma receiving PD-1 inhibitor-based therapy stratified by PD-L1 CPS ≥5 (ABC). PD-L1: programmed death ligand 1; MSI-H: microsatellite instability-high; MSS: microsatellite stability; Mut: mutation; NDB: no durable benefit; OS, overall survival; PFS, progression-free survival; PD-L1: programmed death ligand 1; TMB: tumor mutation burden. Figure S6. The spectrum of top 30 co-occurring or exclusively occurring mutations for MSI-H (A) and MSS (B) cholangiocarcinoma. MSI-H: microsatellite instability-high, MSS: microsatellite stability. Table S7. Baseline clinicopathological characteristics of 139 patients with advanced cholangiocarcinoma who received PD-1 inhibitor-based therapy. [file 12916_2024_3257_MOESM2_ESM.docx]

**Genomic characterization and immunotherapy for microsatellite instability-high in cholangiocarcinoma**

**Additional file 2**

**Table S2.** Clinicopathological characteristics of 887 patients with cholangiocarcinoma

|  | **MSI-H (N=48)** | **MSS (N=839)** | **P-value** | |
| --- | --- | --- | --- | --- |
| Gender |  |  |  |  |
| Male | 29 (60.4) | 505 (60.2) | 1.000 |  |
| Female | 19 (39.6) | 334 (39.8) |  |  |
| Age |  |  |  |  |
| Median (range) | 56 (30-81) | 60 (19-89) | 0.180 |  |
| <60 | 28 (58.3) | 397 (47.3) | 0.141 |  |
| ≥60 | 20 (41.7) | 442 (52.7) |  |  |
| Tumor location |  |  | 0.349 |  |
| ICC | 35 (72.9) | 549 (65.4) |  |  |
| ECC | 13 (27.1) | 290 (34.6) |  |  |
| Largest tumor size |  |  | 0.626 |  |
| <5cm | 22 (45.8) | 368 (43.9) |  |  |
| ≥5cm | 14 (29.2) | 209 (24.9) |  |  |
| UK | 12 (25.0) | 262 (31.2) |  |  |
| Tumor differentiation |  |  | 0.541 |  |
| Well | 0 (0.0) | 14 (1.7) |  |  |
| Middle | 18 (37.5) | 321 (38.3) |  |  |
| Poor | 24 (50.0) | 355 (42.3) |  |  |
| UK | 6 (12.5) | 149 (17.8) |  |  |
| Lymph node metastasis |  |  | **0.002** |  |
| Yes | 23 (47.9) | 244 (29.1) |  |  |
| No | 22 (45.8) | 377 (44.9) |  |  |
| UK | 3 (6.3) | 218 (26.0) |  |  |
| Stage |  |  | 0.061 |  |
| I-II | 18 (37.5) | 339 (40.4) |  |  |
| III-IV | 28 (58.3) | 375 (44.7) |  |  |
| UK | 2 (4.2) | 125 (14.9) |  |  |
| PD-L1 expression |  |  | **<0.001** |  |
| Positive (CPS≥1) | 18 (37.5) | 100 (11.9) |  |  |
| Negative (CPS<1) | 10 (20.8) | 273 (32.5) |  |  |
| UK | 20 (41.7) | 466 (55.5) |  |  |
| TMB |  |  | **<0.001** |  |
| <10 muts/Mb | 0 (0) | 766 (91.3) |  |  |
| ≥10 muts/Mb | 48 (100) | 73 (8.7) |  |  |
| Median TMB (IQR) | 41.7 (32.0-55.1) | 3.1 (1.8-5.6) | **<0.001** |  |

Abbreviations: CPS, combined positive score; ECC, extrahepatic cholangiocarcinoma; ICC, intrahepatic cholangiocarcinoma; IQR, interquartile range; MSI-H, microsatellite instability-high, MSS, microsatellite stability; PD-L1, programmed death ligand 1; TMB, tumor mutation burden; UK, unknow

**
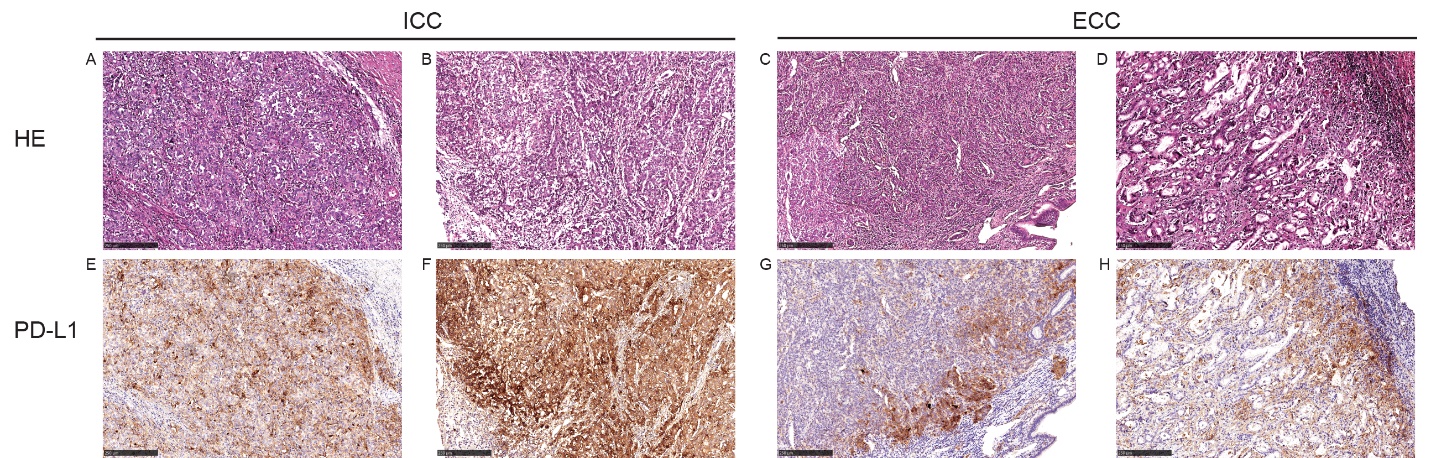
**

**Figure S1.** Representative cases of hematoxylin-eosin staining and high PD-L1 expression of MSI-H patients with cholangiocarcinoma. **(ABEF)** Two ICC cases, **(CDGH)** 2 ECC cases. ECC: extrahepatic cholangiocarcinoma; ICC: intrahepatic cholangiocarcinoma


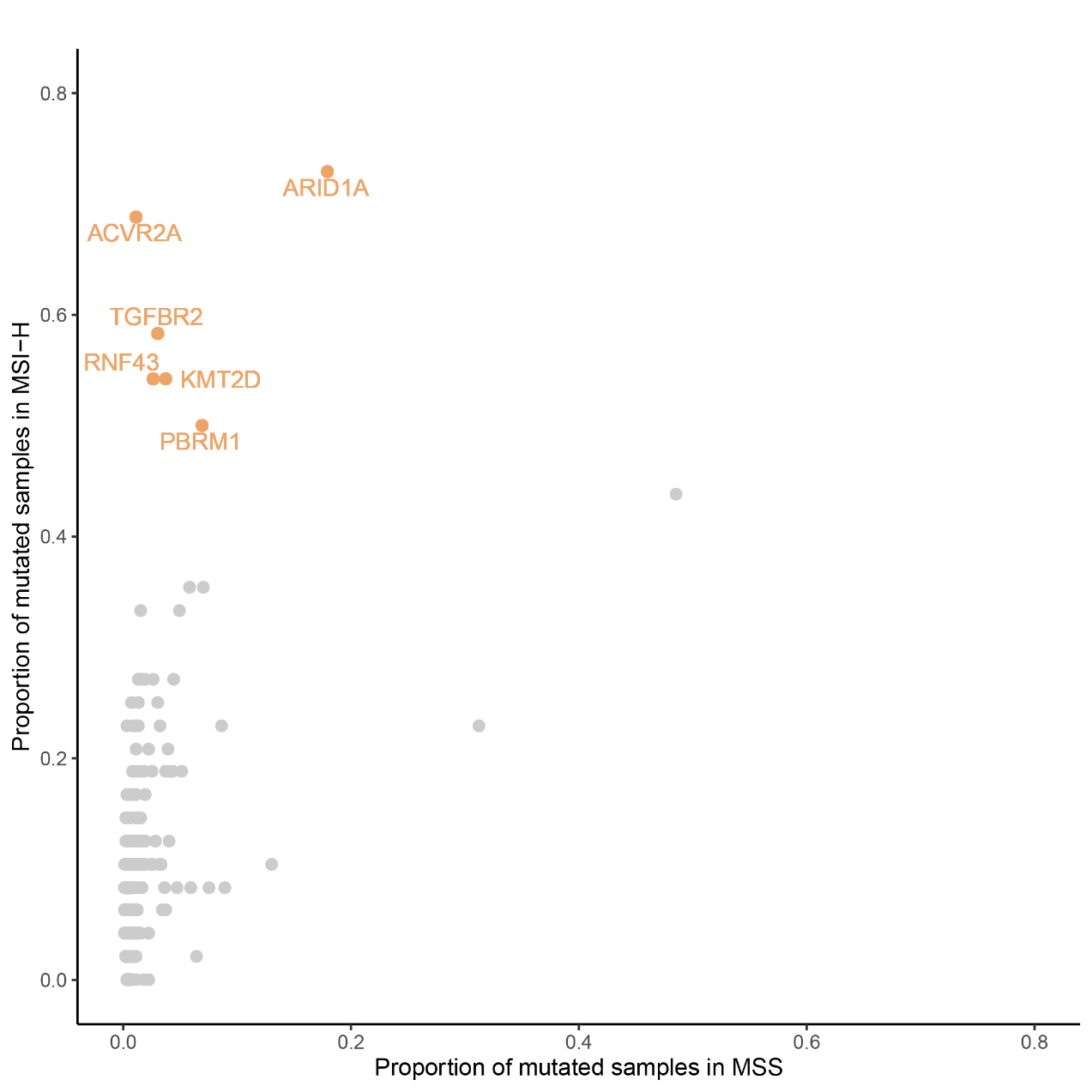


**Figure S2.** Proportion of mutated cholangiocarcinoma samples by MSI status: (y-axis) proportion in MSI-H-specific gene; (x-axis) proportion in MSS-specific gene. MSI-H: microsatellite instability-high, MSS: microsatellite stability


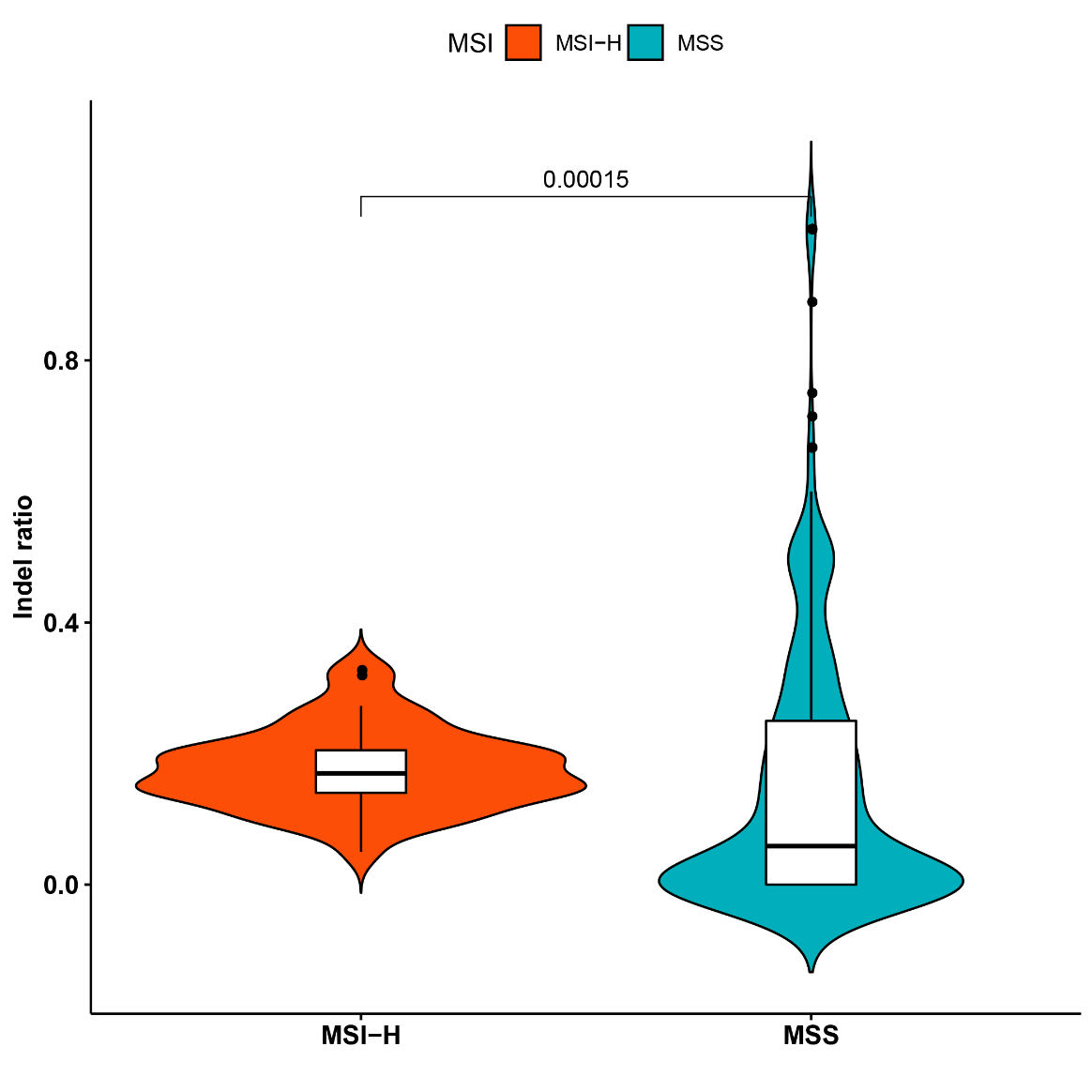


**Figure S3.** The indel ratio of cholangiocarcinoma in MSI-H and MSS cancer. MSI-H: microsatellite instability-high, MSS: microsatellite stability


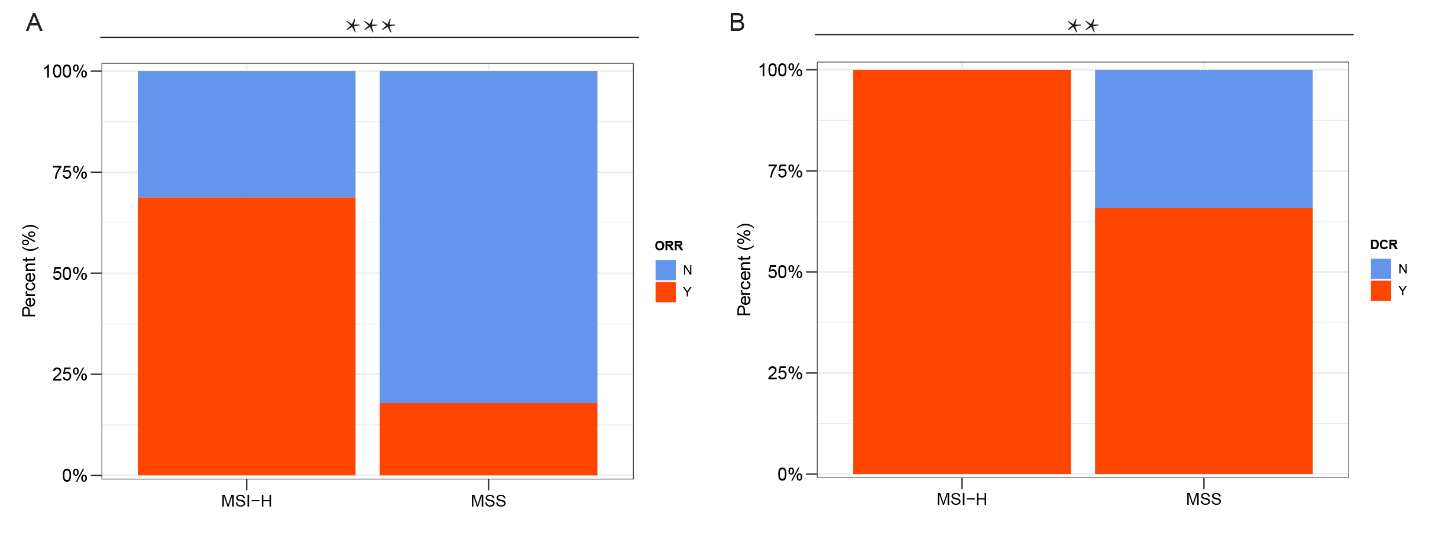


**Figure S4.** ORR **(A)** and DCR **(B)** of 139 advanced cholangiocarcinoma patients receiving PD-1 inhibitor-based therapy stratified by MSI status. DCR: disease control rate, MSI: microsatellite instability, ORR: Objective Response Rate

**
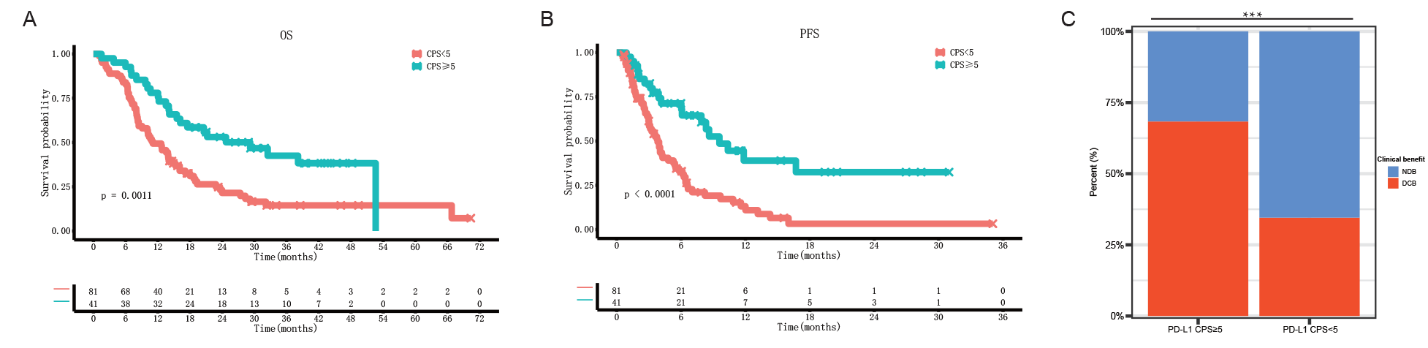
**

**Figure S5.** Kaplan‒Meier estimates of overall survival and progression-free survival and clinical benefit histogram of patients with advanced cholangiocarcinoma receiving PD-1 inhibitor-based therapy stratified by PD-L1 CPS ≥5 **(ABC)**.

PD-L1: programmed death ligand 1; MSI-H: microsatellite instability-high; MSS: microsatellite stability; Mut: mutation; NDB: no durable benefit; OS, overall survival; PFS, progression-free survival; PD-L1: programmed death ligand 1; TMB: tumor mutation burden


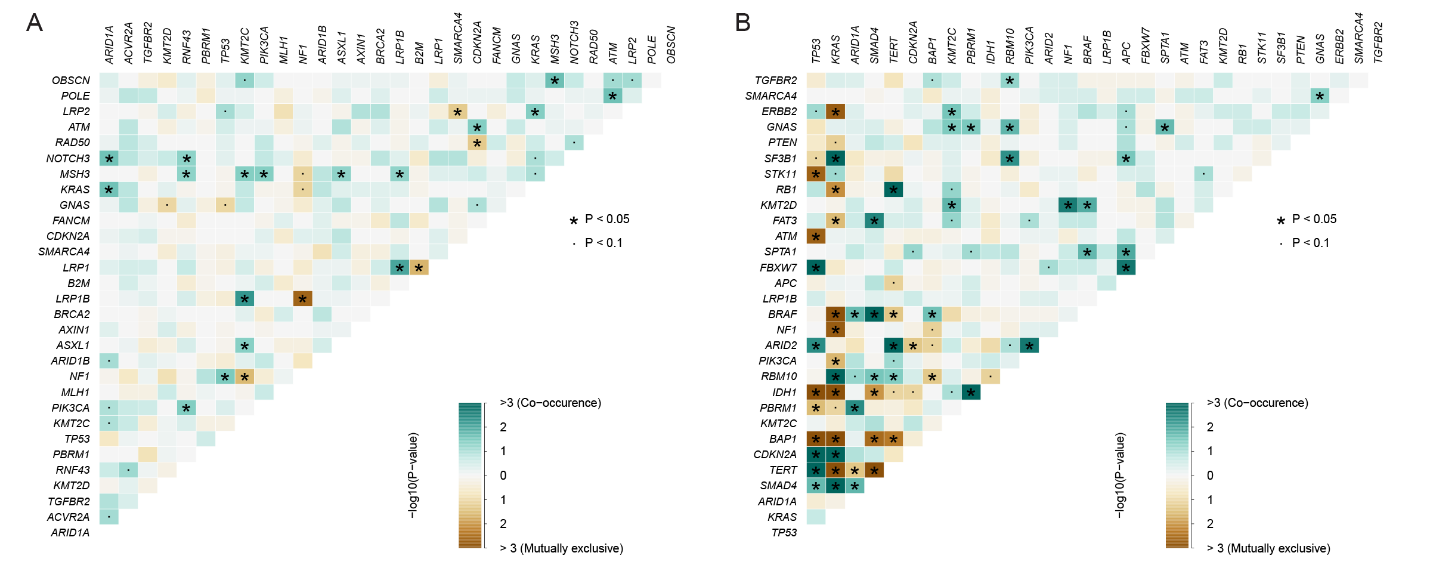


**Figure S6.** The spectrum of top 30 co-occurring or exclusively occurring mutations for MSI-H **(A)** and MSS **(B)** cholangiocarcinoma. MSI-H: microsatellite instability-high, MSS: microsatellite stability

Table S7. Baseline clinicopathological characteristics of 139 patients with advanced cholangiocarcinoma who received PD-1 inhibitor-based therapy.

| **Variable** | **MSI-H/dMMR (N=16)** | **MSS (N=123)** | **P value** | **All**  **(N=139)** |
| --- | --- | --- | --- | --- |
| Median age (range) | 48.5 (30-70) | 61.0 (33-84) | **0.003** | 61 (30-84) |
| <60 year | 11 (68.8) | 52 (42.3) | 0.061 | 63 (45.3) |
| ≥60 year | 5 (31.3) | 71 (57.7) |  | 76 (54.7) |
| Gender — no. (%) |  |  | 0.429 |  |
| Male | 11 (68.8) | 70 (56.9) |  | 81 (58.3) |
| Female | 5 (31.5) | 53 (43.1) |  | 58 (41.7) |
| Tumor location— no. (%) |  |  | 0.203 |  |
| ICC | 10 (62.5) | 97 (78.9) |  | 107 (77.0) |
| ECC | 6 (37.5) | 26 (21.1) |  | 32 (23.0) |
| Adenocarcinoma— no. (%) | 16 (100.0) | 123 (100.0) | 1.000 | 139 (100.0) |
| Differentiation (low) — no. (%) | 8 (50.0) | 51 (41.5) | 0.595 | 59 (42.4) |
| Biliary stone disease— no. (%) | 2 (12.5) | 22 (17.9) | 0.739 | 24 (17.3) |
| Child–Pugh Grade—no. (%) |  |  |  |  |
| A | 16 (100) | 114 (92.7) | 0.598 | 130 (93.5) |
| B | 0 | 9 (7.3) |  | 9 (6.5) |
| Largest tumor size — no. (%) |  |  | 0.280 |  |
| <5 cm | 12 (75.0) | 72 (58.5) |  | 84 (60.4) |
| ≥5 cm | 4 (25.0) | 51 (41.5) |  | 55 (39.6) |
| T stage— no. (%) |  |  | 0.881 |  |
| T1-T2 | 8 (50.0) | 66 (53.7) |  | 74 (53.2) |
| T3-T4 | 5 (31.3) | 30 (24.4) |  | 35 (25.2) |
| UK | 3 (18.8) | 27 (22.0) |  | 30 (21.6) |
| N stage— no. (%) |  |  | 0.263 |  |
| N0 | 4 (25.0) | 28 (22.8) |  | 32 (23.0) |
| N1 | 9 (56.3) | 84 (68.3) |  | 93 (66.9) |
| N2 | 2 (12.5) | 4 (3.3) |  | 6 (4.3) |
| UK | 1 (6.3) | 7 (5.7) |  | 8 (5.8) |
| M stage— no. (%) |  |  | 0.069 |  |
| M0 | 11 (68.8) | 54 (43.9) |  | 65 (46.8) |
| M1 | 5 (31.3) | 69 (56.1) |  | 74 (53.2) |
| TNM stage— no. (%) |  |  | **0.028** |  |
| I | 2 (12.5) | 2 (1.6) |  | 4 (2.9)* |
| II | 1 (6.3) | 15 (12.2) |  | 16 (11.5) |
| III | 8 (50.0) | 37 (30.1) |  | 45 (32.4) |
| IV | 5 (31.3) | 69 (56.1) |  | 74 (53.2) |
| Previous surgery resection | 10 (62.5) | 65 (52.8) | 0.596 | 75 (54.0) |
| Numbers of prior systemic  therapies— no. (%) |  |  | 0.167 |  |
| 0 | 13 (81.3) | 74 (60.2) |  | 87 (62.6) |
| 1 | 1 (6.3) | 34 (27.6) |  | 35 (25.2) |
| ≥2 | 2 (12.5) | 15 (12.2) |  | 17 (12.2) |
| ECOG-PS — no. (%) |  |  | **0.036** |  |
| 0 | 9 (56.3) | 35 (28.5) |  | 44 (31.7) |
| 1 | 7 (43.8) | 85 (52.8) |  | 72 (51.8) |
| 2 | 0 (0.0) | 23 (18.7) |  | 23 (16.5) |
| PD-1 inhibitor based therapy— no. (%) |  |  | **0.008** |  |
| Target therapy | 9 (56.3) | 106 (86.2) |  | 115 (82.7) |
| Chemotherapy | 7 (43.8) | 17 (13.8) |  | 24 (17.3) |
| PD-L1 expression — no. (%) |  |  | **0.005** |  |
| CPS<1 | 3 (18.8) | 57 (46.3) |  | 60 (43.2) |
| 1≤CPS<5 | 0 (0) | 21 (17.1) |  | 21 (15.1) |
| CPS≥5 | 10 (62.5) | 31 (25.2) |  | 41 (29.5) |
| UK | 3 (18.8) | 14 (11.4) |  | 17 (12.2) |
| TMB — no. (%) |  |  | **0.002** |  |
| <10 muts/Mb | 0 (0.0) | 108 (87.8) |  | 108 (77.7) |
| ≥10 muts/Mb | 12 (75.0) | 15 (12.2) |  | 27 (19.4) |
| UK | 4 (25.0) | 0 (0.0) |  | 4 (2.9) |
| SWI/SNF pathway— no. (%) |  |  | **<0.001** |  |
| Mut | 12 (75.0) | 37 (30.1) |  | 49 (35.3) |
| WT | 0 (0.0) | 86 (69.9) |  | 86 (61.9) |
| UK | 4 (25.0) | 0 (0.0) |  | 4 (2.9) |
| MMR pathway— no. (%) |  |  | **<0.001** |  |
| Mut | 10 (62.5) | 8 (6.5) |  | 18 (12.9) |
| WT | 2 (12.5) | 115 (93.5) |  | 117 (84.2) |
| UK | 4 (25.0) | 0 (0.0) |  | 4 (2.9) |
| WNT pathway— no. (%) |  |  | **<0.001** |  |
| Mut | 9 (56.3) | 21 (17.1) |  | 30 (21.6) |
| WT | 3 (18.8) | 102 (82.9) |  | 105 (75.5) |
| UK | 4 (25.0) | 0 (0.0) |  | 4 (2.9) |
| Response — no. (%) |  |  | **<0.001** |  |
| PR | 11 (68.8) | 22 (17.9) |  | 33 (23.7) |
| SD | 5 (31.3) | 59 (48.0) |  | 64 (46.0) |
| PD | 0 (0) | 42 (34.1) |  | 42 (30.2) |
| CBR — no. (%) |  |  | **<0.001** |  |
| DCB | 14 (87.5) | 51 (41.5) |  | 65 (46.8) |
| NDB | 2 (12.5) | 72 (58.5) |  | 74 (53.2) |
| PFS | NE | 4.0 (2.7-5.3) | HR=0.14 (95%CI: 0.05-0.34, P<0.001) | 5.3 (3.7-6.9) |
| OS | NE | 13.5 (10.3-16.8) | HR=0.17 (95%CI: 0.06-0.46, P=0.001) | 14.8 (12.6-16.9) |

Abbreviations: CBR, clinical benefit response; CI, confidence interval; CPS, combined positive score; DCB, Durable clinical benefit; dMMR, deficient DNA mismatch repair; ECC, extrahepatic cholangiocarcinoma; ECOG-PS, Eastern Cooperative Oncology Group-performance status; HR, hazard ratio; ICC, intrahepatic cholangiocarcinoma; MMR, Mismatch Repair; MSI-H, microsatellite instability-high, MSS, microsatellite stability; Mut: Mutation; NDB, no durable benefit; NE, not evaluable; OS, overall survival; PD, Progressive disease; PFS, progression-free survival; PD-L1, programmed death ligand 1; PR, partial response; SD, stable disease; SWI/SNF, Switch/Sucrose nonfermentable; TMB, tumor mutation burden; UK, unknow; WNT, Wingless/Integrated; WT: wild type.

*: Local recurrence after resection
